# Vascular nitric oxide resistance in type 2 diabetes

**DOI:** 10.1038/s41419-023-05935-5

**Published:** 2023-07-11

**Authors:** Zahra Bahadoran, Parvin Mirmiran, Khosrow Kashfi, Asghar Ghasemi

**Affiliations:** 1 Nutrition and Endocrine Research Center, Research Institute for Endocrine Sciences, Shahid Beheshti University of Medical Sciences, Tehran, Iran; 2 Department of Clinical Nutrition, Faculty of Nutrition Sciences and Food Technology, National Nutrition and Food Technology Research Institute, Shahid Beheshti University of Medical Sciences, Tehran, Iran; 3 Department of Molecular, Cellular, and Biomedical Sciences, Sophie Davis School of Biomedical Education, City University of New York School of Medicine, New York, NY 10031 USA; 4 Endocrine Physiology Research Center, Research Institute for Endocrine Sciences, Shahid Beheshti University of Medical Sciences, Tehran, Iran

**Keywords:** Valvular disease, Diabetes

## Abstract

Vascular nitric oxide (NO•) resistance, manifested by an impaired vasodilator function of NO• in both the macro- and microvessels, is a common state in type 2 diabetes (T2D) associated with developing cardiovascular events and death. Here, we summarize experimental and human evidence of vascular NO• resistance in T2D and discuss its underlying mechanisms. Human studies indicate a ~ 13-94% decrease in the endothelium (ET)-dependent vascular smooth muscle (VSM) relaxation and a 6-42% reduced response to NO• donors, i.e., sodium nitroprusside (SNP) and glyceryl trinitrate (GTN), in patients with T2D. A decreased vascular NO• production, NO• inactivation, and impaired responsiveness of VSM to NO• [occurred due to quenching NO• activity, desensitization of its receptor soluble guanylate cyclase (sGC), and/or impairment of its downstream pathway, cyclic guanosine monophosphate (cGMP)-protein kinase G (PKG)] are the known mechanisms underlying the vascular NO• resistance in T2D. Hyperglycemia-induced overproduction of reactive oxygen species (ROS) and vascular insulin resistance are key players in this state. Therefore, upregulating vascular NO• availability, re-sensitizing or bypassing the non-responsive pathways to NO•, and targeting key vascular sources of ROS production may be clinically relevant pharmacological approaches to circumvent T2D-induced vascular NO• resistance.

## Facts

- Current evidence implies on the presence of vascular nitric oxide (NO•) resistance, an independent risk factor for cardiovascular events, in patients with type 2 diabetes (T2D).
- Vascular NO• resistance in T2D is stage-dependent and displays a progressive spectrum, initially manifested by an augmented or preserved vascular NO• production and/or vascular smooth muscle (VSM) response to NO•, followed by a reduced NO• bioavailability and/or partial to almost entirely impaired NO• function in VSM, in both the macro- and microvessels.
- Quenching NO• activity, desensitization of soluble guanylate cyclase (sGC), and/or impairment of cyclic guanosine monophosphate (cGMP)-protein kinase G (PKG) are the known mechanisms underlying the vascular NO• resistance in T2D.

## Questions

- Which mechanism(s) is/are initiator(s) and key player(s) in developing vascular NO• resistance in T2D?
- Do different vessels display diverse phenotypes of vascular NO• resistance?
- Which vessels are more sensitive to and affected earlier by NO• resistance in T2D?
- Which pharmaceutical approaches could be effective in preventing and retarding the progression of T2D-induced vascular NO• resistance?

## Introduction

The global prevalence of diabetes in adults was reported to be 10.5% (536.6 million people) in 2021, reaching up to 12.2% (783.2 million) in 2045 [1]. Type 2 Diabetes (T2D), accounting for approximately 90% of diabetes cases, is related to macrovascular and microvascular complications, with an overall prevalence of 32.2% and 12.0%, respectively [2, 3]. In addition, cardiovascular disease (CVD) mortality is estimated to constitute 50.3% of all deaths in patients with T2D [2]. Both endothelium (ET) and vascular smooth muscle (VSM) layer are functionally impaired in diabetic vessels [4, 5], and vascular complications account for the most significant part of diabetes-associated morbidity and mortality [6].

Nitric oxide (NO•) is the most critical vasodilator produced by vascular ET [7]. Hyperglycemia-induced overproduction of reactive oxygen species (ROS) in T2D, which triggers several biochemical pathways [i.e., polyol and hexosamine pathway, advanced glycation end products (AGEs) production, activation of protein kinase C (PKC) and its downstream targets, especially nicotinamide adenine dinucleotide phosphate (NADPH) oxidase (NOX)], and vascular insulin resistance result in impaired metabolism of NO• (a crucial player in vascular homeostasis) and development of vascular dysfunction [8, 9].

The NO• resistance syndrome, a state of decreased NO• production by the ET [10–12], enhanced NO• inactivation [10–12], and impaired responsiveness to NO• at receptor level or its subsequent signal transduction [10–15], has been documented in the VSM in T2D [11, 14, 16–18]. Furthermore, the vascular NO• resistance presented as impaired vasodilator function of NO• manifests in both the macro- [i.e., large elastic and muscular arteries] [19–21] and micro- [i.e., vessels with a diameter <150 μm, including arterioles and venules] [22–25] vessels. The vascular NO• resistance is associated with future cardiovascular events (i.e., myocardial infarction, definite angina, coronary revascularization, stroke, resuscitated cardiac arrest, and CVD mortality), independent of the other well-known risk factors [26, 27]. Here, we summarize evidence of vascular NO• resistance from animal and human studies and discuss the underlying mechanisms of vascular NO• resistance in T2D.

## Evidence of vascular NO• resistance in T2D

### Human studies

As shown in Table 1, impairment of ET-dependent VSM relaxation has been consistently reported in both prediabetes [23, 28–30] and established T2D [12, 22, 23, 25, 28–47]. Impairment of ET-dependent VSM relaxation in T2D has been documented mostly in the brachial artery [11, 12, 30–45] and to a lesser extent in thoracic [10, 19, 46] and femoral [28, 29] arteries, as well as in saphenous vein [46] and skin microvessels [22, 23, 25, 48]. To assess ET-dependent VSM relaxation, flow-mediated dilatation (FMD) [34], a noninvasive technique for measuring NO•-mediated vascular function [49], as well as infusion of serotonin [40] and cholinergic agonists, mainly acetylcholine (ACh) [22, 23, 25, 31–33, 35–39, 41, 42, 44–47] and methacholine [28, 29], have been used in different studies. Overall, results indicate a ~ 13-94% decrease in ET-dependent VSM relaxation in T2D patients. ET-independent, NO•-dependent relaxation has been reported to be decreased in some prediabetes subjects by 26-33% [23, 30], but it preserves in others [28, 29]. The same is true in the case of established T2D, where responses to NO• donors, sodium nitroprusside (SNP), and glyceryl trinitrate (GTN) are decreased by 6-42% [21–23, 25, 31–33, 36, 37, 40, 41, 43–45, 47] or preserved [19, 28, 29, 34, 35, 38, 39, 42, 50].

**Table 1:** Impaired endothelium (ET)-dependent and ET-independent, nitric oxide (NO•)-dependent vascular smooth muscle (VSM) relaxation in patients with type 2 diabetes (T2D) or impaired glucose and insulin homeostasis.

Study	Condition	Age (years)	Duration of T2D (years)	HbA1C (%)	Vessel type	ET-dependent VSM relaxation	ET-independent, NO•-dependent VSM relaxation
Steinberg et al. [28]	Obese-IR	35	–	NR	Femoral a.	↓Meth (40%)	↔SNP
Steinberg et al.^a^ [29]	Obese-IR	34	–	NR	Femoral a.	↓Meth (52%)	↔SNP
Steinberg et al.^b^ [29]	Obese-IR	34	–	NR	Femoral a.	↓Meth (41%)	↔SNP
Caballero et al. [23]	IGT	50	–	5.7	FSM	↓ACh (23%)	↓SNP (26%)
Sivitz et al. [30]	IFG	56	–	7.6	Brachial a.	↓ACh (31%)	↓SNP (33%)
McVeigh et al. [31]	T2D	53	5.2	NR	Brachial a.	↓ACh (94%)	↓GTN (32%)
Ting et al. [12]	T2D	47	3.5	7.9	Brachial a.	↓Meth (37%)	NR
Watts et al. [32]	T2D	55	3.6	NR	Brachial a.	↓ACh (52%)	↓SNP (25%)
Goodfellow et al. [50]	T2D	50	3.8	9.7	Brachial a.	NR	↔GTN
Hogikyan et al. [33]	T2D	57	5.6	10.3	Brachial a.	↓ACh (50%)	↓SNP (21%)
Enderle et al. [34]	T2D	57	7.4	9.1	Brachial a.	↓FMD (50%)	↔GTN
Mäkimattila et al. [35]	T2D	51	3.5	NR	Brachial a.	↓ACh (37%)	↔SNP
Gazis et al. [36]	T2D	57	4.6	6.9	Brachial a.	↓ACh (28%)	↓SNP (10%)
Preik et al. [37]	T2D	60	10.0	9.6	Brachial a.	↓ACh (41%)	↓SNP (6%)
Heitzer et al. [38]	T2D	52	5.3	7.8	Brachial a.	↓ACh (47%)	↔SNP
Kimura et al. [39]	T2D	70	7.0	8.0	Brachial a.	↓ACh (31%)	↔GTN
van Etten et al. [40]	T2D	58	NR	NR	Brachial a.	↓Ser (50%)	↓SNP (30%)
Vehkavaara and Yki-Järvinen [41]	T2D	59	> 3.0	9.1	Brachial a.	↓ACh (28%)	↓SNP (15%)
Ifrim and Vasilescu et al. [42]	T2D	56	5.6	9.2	Brachial a.	↓ACh (20%)	↔GTN
Natali et al. [43]	T2D	56-58	5-8	7.6-8.1	Brachial a.	↓ACh	↓SNP
Woodman et al. [44]	T2D	55	NR	NR	Brachial a.	↓ACh (44%)	↓SNP (33%)
Sivitz et al. [30]	T2D	56	NR	5.9	Brachial a.	↓ACh (42%)	↓SNP (42%)
Steinberg et al. [28]	T2D	40	NR	NR	Femoral a.	↓Meth (55%)	↔SNP
Steinberg et al.^a^ [29]	T2D	39	NR	NR	Femoral a.	↓Meth (40%)	↔SNP
Steinberg et al.^b^ [29]	T2D	36	NR	NR	Femoral a.	↓Meth (70%)	↔SNP
Karasu et al.^a^ [46]	T2D	55	11.0	NR	Thoracic a.	↓ACh (54%)	↔SNP
Tawa et al. [19]	T2D	73	7.1	7.0	Thoracic a.	NR	↔GTN
Karasu et al.^a^ [46]	T2D	55	11.0	NR	Saphenous v.	↓ACh (62%)	↔SNP
Caballero et al. [23]	T2D	53	4.3	8.0	FSM	↓ACh (29%)	↓SNP (33%)
Morris et al.^a^ [22]	T2D	59	9.1	6.5	FSM	↓ACh (38%)	↓SNP (21%)
Beer et al. [25]	T2D	58	10.8	8.2	FSM	↓ACh (15%)	↓SNP (21%)
Brooks et al. [47]	T2D	57	9.9	6.7	FSM	↓ACh (44%)	↓SNP (47%)
Natali et al. [45]	T2D	59	5.0	8.0	Brachial a.	↓ACh (52%)	↓SNP (27%)
Vavuranakis et al. [21]	T2D	53	4.2	9.1	Coronary a.	NR	↓GTN (29%)
Preik et al. [37]	T2D	57	9.0	9.8	Brachial a.	↓ACh (58%)	↓SNP (42%)
Beer et al. [25]	T2D	54	6.4	7.3	FSM	↓ACh (13%)	↓SNP (16%)
Brooks et al. [47]	T2D	58	10.2	8.3	FSM	↓ACh (23%)	↓SNP (32%)

Data are reported for both sexes otherwise indicated (^a^men, ^b^women).

*a* artery, *v* vein, *ACh* acetylcholine, *FMD* flow-mediated dilatation, *FSM* forearm skin microcirculation, *GTN* glyceryl trinitrate, *HbA1C* glycated hemoglobin, *IFG* impaired fasting glucose, *IGT* impaired glucose tolerance, *IR* insulin resistance, *Meth* methacholine, *Ser* serotonin, *SNP* sodium nitroprusside, *NR* not reported.

A meta-analysis of published data evaluating ET-dependent VSM relaxation and ET-independent, NO•-dependent VSM relaxation in T2D patients compared with aged-matched controls reported a significantly impaired ET-dependent and-independent vascular functions [standardized mean difference (SMD)= −0.89 and −0.69) that were stronger in micro rather macrocirculation [5].

Likely explanations for discrepancy in the presented results are the presence of other comorbidities, such as dyslipidemia [23, 32] or diabetes complications [51], including neuropathy [52], severity and duration of T2D [45], and good management of the T2D to meet the therapeutic targets [20]. These factors may affect the vessel response to nitrovasodilators [20, 23, 32, 35, 44] and are estimated to account for about 32-37% of the variation in the VSM response to NO• [23]. Furthermore, impaired vasodilatory response to ACh in patients with T2D is negatively correlated with serum triglycerides and positively correlated with high-density lipoprotein-cholesterol [23, 32]. On the other hand, ET-dependent and—independent vascular functions are preserved in the brachial artery of complication-free T2D patients [51]. In addition, SNP-induced vasodilatation was significantly reduced only in the neuropathic T2D patients, compared to either the non-neuropathic diabetic or the non-diabetic controls. In contrast, ACh-induced vasodilation was comparable with non-neuropathic diabetic patients [52].

Some evidence supports the notion that vascular NO• resistance depends on the disease’s severity and treatment modality. Decreased ET-dependent VSM relaxation has been reported to be 22%, 40%, and 52% in insulin-sensitive, intermediate insulin-resistant, and insulin-resistant T2D patients, respectively [45]; in this study, decreased ET-independent, NO•-dependent VSM relaxation was 3%, 7%, and 27%, respectively [45]. In addition, in people with T2D who had glycated hemoglobin (HbA1C)<7%, skeletal muscle ET function was similar to non-diabetic subjects, whereas it was lower by ~30% in T2D patients with HbA1C > 7% [20]. Insulin therapy can significantly affect nitrovasodilatory response in T2D; a 6-month and a 3.5-y follow-up of T2D patients indicated that insulin therapy resulted in ACh- and SNP-induced vasodilation to return to normal levels and even higher than the controls [41].

### Animal studies

As shown in Table 2, an impaired ET-dependent VSM relaxation has consistently been documented in established T2D [53–64], whereas for the prediabetes state, both preserved [59, 63–65] and decreased (20–89%) [59, 64, 66–68] ET-dependent VSM relaxation have been reported. The impaired ET-dependent VSM relaxation in T2D has been reported mostly in mesenteric arteries (5–66%) [53, 55–58, 60–62, 64]; however, it has also been documented in other vessels, including the aorta [59, 64], pulmonary [59], thoracic [62], coronary [64], femoral [63], and uterine [54] arteries. ET-independent, NO•-dependent VSM relaxation has mainly been preserved [64, 66–68] or upregulated [64, 65] in prediabetes. The augmented or preserved VSM response to NO• in prediabetes may be attributed to increased compensatory NO• production and/or increased sensitivity and activity of VSM soluble guanylate cyclase (sGC). In the case of established T2D, ET-independent, NO•-dependent VSM relaxation is mainly preserved in mesenteric [53, 55, 56, 58, 60–62, 64], thoracic [62], coronary [64], and femoral [63] arteries; however, a decreased VSM response to NO• has been reported in mesenteric [57] and uterine arteries [54].

**Table 2:** Impaired endothelium (ET)-dependent and ET-independent, nitric oxide (NO•)-dependent vascular smooth muscle (VSM) relaxation in animal models with prediabetes and type 2 diabetes (T2D).

Study	Model/species	Vascular region assessed	ET-dependent VSM relaxation	ET-independent, NO•-dependent VSM relaxation
*Prediabetes*				
Oltman et al. [64]	Zucker obese rats	Aorta	↔ ACh	↔ SNP
Oltman et al. [64]	Zucker obese rats	Mesenteric a.	↔ ACh	↑ SNP (56%)
Mourmoura et al. [65]	Zucker obese rats	Coronary a.	↔ ACh	↑ SNP (19%)
Lu et al. [63]	Zucker obese rats	Femoral a.	↔ ACh	NR
Melo et al. [59]	HFD-fed rats	Pulmonary a.	↔ ACh	NR
Melo et al. [59]	HFD-fed rats	Aorta	↓ ACh (44%)	NR
Belin et al. [66]	Zucker obese rats	Mesenteric a.	↓ ACh (20%)	↔ SNP
Qiu et al. [68]	*db/db* obese mice	Mesenteric a.	↓ ACh (46%)	↔ SNP
Oltman et al. [64]	Zucker obese rats	Coronary a.	↓ ACh (89%)	↔ SNP
*Stablished T2D*				
Mishra et al. [56]	Goto-Kakizaki rats	Mesenteric a.	↓ ACh (34%)	↔ SNP
Belin et al. [61]	Zucker obese HFD-fed rats	Mesenteric a.	↓ ACh (44%)	↔ SNP
Wang et al. [62]	Zucker obese rats	Mesenteric a.	↓ ACh (13%)	↔ SNP
Oltman et al. [64]	Zucker obese rats	Mesenteric a.	↓ ACh (25%)	↔ SNP
Pannirselvam et al. [58]	*db/db* obese mice	Mesenteric a.	↓ ACh (34%)	↔ SNP
Leo et al. [60]	Zucker obese rats	Mesenteric a.	↓ ACh (5%)	NR
Oniki et al. [57]	Goto-Kakizaki rats	Mesenteric a.	↓ ACh (66%)	↓ SNP (71%)
Melo et al. [59]	HFD + HSD-fed rats	Aorta	↓ ACh (56%)	NR
Oltman et al. [64]	Zucker obese rats	Aorta	↓ ACh (12%)	NR
Melo et al. [59]	HFD + HSD-fed rats	Pulmonary a.	↓ ACh (34%)	NR
Wang et al. [62]	Zucker obese rats	Thoracic a.	↓ ACh (29%)	↔ SNP
Oltman et al. [64]	Zucker obese rats	Coronary a.	↓ ACh (70%)	↔ SNP
Lu et al. [63]	Zucker obese rats	Femoral a.	↓ ACh (15%)	↔ SNP
Goulopoulou et al. [54]	Goto-Kakizaki rats^a^	Uterine a.	↓ ACh (14%)	↓ SNP (11%)

All studies were conducted in males except one that has been identified (^a^Female).

*a* artery, *ACh* acetylcholine, *HFD* high-fat diet, *HSD* high-sucrose diet, *SNP* sodium nitroprusside, *NR* not reported.

## Vascular NO• production in normal conditions

All three layers of the vessel wall (i.e., tunica intima, tunica media, and tunica adventitia) and its supportive components, including perivascular adipose tissue (pVAT), nerve fibers, mast cells, and macrophages, contribute to the bioavailable pool of NO• within the vessel wall [69]. In addition, NO• may penetrate the vascular wall from the vessel lumen; red blood cells (RBCs) and other circulating cells (i.e., monocytes, neutrophils, lymphocytes, and platelets) contribute to the circulating pool of NO• reaching the vessel wall [70].

Endothelial NO• synthase (eNOS), neuronal NOS (nNOS), and inducible NOS (iNOS) are differently localized and involved in NO• production within the hierarchy (i.e., tree-like hierarchical branching structure, from larger to smaller branches) of blood vessels in different organs [71, 72]. Traditionally, eNOS was considered the primary source of bioavailable vascular NO•; this view has been changed by studying the genetically-modified animal models (lacking either nNOS or eNOS) demonstrated that nNOS is as important as eNOS in arteriolar relaxation [73] or those indicated that nNOS is the predominant source of NO• in the microvasculature of eNOS-lacking animals [74]. Evidence of iNOS expression in vessels under normal conditions is controversial [72, 75, 76]; however, it is significantly expressed under inflammatory conditions in all three layers of the vessels [75]. The vascular ET expresses all NOS isoforms. ET-derived NO• is released abluminal toward VSMCs and, to a lesser extent, as a spillover into the blood at its luminal side [77]. About 40-60% whole-body NO• production in the human body [~411–661 of 1100 μmol/day [78]] is produced by endothelial cells (ECs) [79]. More than 60% of ET-derived NO• in the vessel wall is detectable in VSMC after about 20 seconds [80].

Human VSMCs express all NOS isoforms [72], however, nNOS seems to be the predominant isoform in VSMCs. The physiologically-relevant vasodilatory role of VSM nNOS-derived NO• was confirmed by evidence showing that potassium chloride (KCl)-induced contraction was significantly elevated in the de-endothelialized aortic rings of nNOS knockout mice compared to controls (151% *vs*. 131% of reference KCl contraction) [81]. The nNOS-derived NO• is involved in autoregulating local vascular tone via direct effects on VSM [82].

pVAT significantly expresses eNOS, which produces measurable amounts of NO• [83]. pVAT-originated NO• seems directly targets VSMCs to induce vessel relaxation via the sGC-cyclic guanosine monophosphate (cGMP)-protein kinase G (PKG) [84]. The attributable effect size of pVAT on ACh-induced relaxation of the aorta has been estimated to be ~40% [85]. The effect is entirely mediated by NO•, given that it was fully blocked by *N*^G^-Methyl-l-arginine (*L*-NMMA) [85]. pVAT-derived NO• is suggested to travel to the tunica media or even the intima and modulate vascular function [86]. The contribution of the adventitia and other components of the vessel wall in vascular NO• availability is less documented. The adventitia seems to be a potent source of NO• than VSMC under an inflammatory condition [87]. Supplementary Table 1 summarizes available data regarding the expression/activity NOS isoforms in different vessels in humans and animals.

Circulating or blood-born NO• [i.e., estimated to be ~15-38 µM [7, 88, 89], derived from RBC-eNOS, free NO•, nitrate (NO_3_^–^), NO_2_^–^, *S*-nitrosothiols (SNOs), *S*-nitrosoalbumin (SNO-albumin), and SNO-hemoglobin (Hb-SNO)] may significantly contribute to the vascular NO• pool [77, 90–92]. In macro- and micro-vessels, the contribution of circulating NO• to available NO• within the vessel wall is estimated about 60% [93] and 7%, respectively. A small fraction (~3.4 ± 0.58 nM) of circulating NO• [94], diffuses through RBC-free zone of laminar flowing blood and can be transported into the vascular wall [95]. RBC-eNOS produces 4.6 µmol NO• per day [90], and that NO• has an essential and independent contribution to VSM relaxation [77]. NO• delivered by cell-free Hb and Hb-SNO to the vascular wall is estimated ~0.02 and 0.25-6 pM [96, 97], respectively. SNO-albumin releases NO• ~1.4 pmol NO•/min [88].

## Vascular NO• production in T2D

Changes in whole-body NO• production in T2D depend on the disease’s duration. An enhanced NO• production occurs in the initial stages of T2D; this idea is supported by several lines of evidence, e.g., an elevated serum NO• concentration in T2D patients at the initial stages (5 years of the onset), compared to its reduced level in patients with prolonged T2D [98]. On the other hand, a decreased whole-body [99, 100] and vascular NO• production [101, 102] have been documented in established T2D. A reduced fractional synthesis rate (FSR) of NO• (i.e., percent of circulating pool newly synthesized from l-arginine) (19.3 ± 3.9% vs. 22.9 ± 4.5% per day) and absolute synthesis rate (ASR) of NO• (320 vs. 890 μmol per day) were observed in patients with established T2D compared to normal subjects [99]. In addition, a 50% decreased NO• production from plasma l-arginine turnover (0.009 ± 0.002 vs. 0.019 ± 0.007 µmole/kg/h, 0.19% vs. 0.35%), and a 16% decrease of the total NO• synthesis rate (0.52 ± 0.16 vs. 0.62 ± 0.16 µmole/kg/h) have been observed in established T2D compared to healthy subjects [100].

Decreased NO• production in diabetes is attributed to diminished eNOS expression [99], overexpression of negative regulators of eNOS activity [i.e., the membrane-associated scaffolding protein caveolin-1 (Cav-1) and phosphatase and tensin homolog (PTEN)] [103–105], substrate deficiency for NOSs [due to increased arginase activity and decreased l-arginine availability [106, 107]], eNOS uncoupling [due to elevated ROS, oxidation of tetrahydrobiopterin (BH_4_), l-arginine depletion, and accumulation of methylarginines] resulting in superoxide anion instead of NO production [108].

**Fig. 1:**
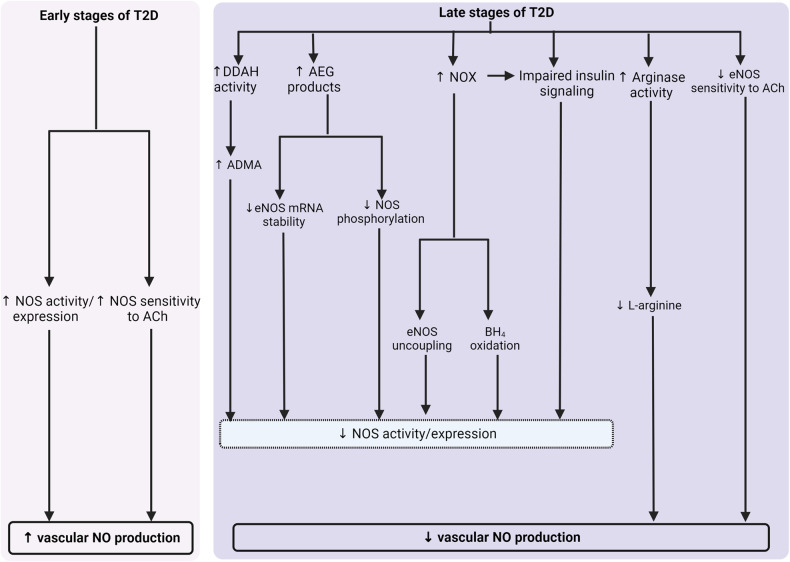
Increased and decreased vascular nitric oxide (NO•) production in the early and late stages of type 2 diabetes (T2D), respectively. ACh, acetylcholine; ADMA, asymmetric dimethyl arginine; AEG, advanced end-glycation products; BH_4_, tetrahydrobiopterin; DDAH, dimethylarginine dimethylaminohydrolase; NOS, NO• synthase; NOX, nicotinamide adenine dinucleotide phosphate (NADPH) oxidase.

Changes in vascular NO• levels in T2D are also dependent on the duration of the disease, with increased production in initial stages and decreased production in later stages (Fig. 1); initial stages of T2D may induce compensatory mechanisms, leading to adaptation and relative normalization of NO• vascular output, whereas a more extended duration towards established T2D results in a state of vascular NO• deficiency. In Goto-Kakizaki (GK) rats, ACh-induced NO• release (in the thoracic aorta) was enhanced at 12 weeks but decreased at 36 weeks [109]. At the initial stages of T2D in GK rats (17-week old, in the presence of hyperglycemia and hyperinsulinemia), basal NO• bioavailability in the abdominal aorta was more likely to be elevated compared with aged-matched controls [110]. In streptozotocin (STZ)-induced diabetic rats, the aorta showed significantly elevated NO• levels after 3 weeks of diabetes onset, which remained high after 7 weeks [111]. The mesenteric arteries pVAT has also been reported to undergo an adaptive NO• overproduction (by 2-fold compared to controls) during the initial stages of insulin resistance (8-week high-fat diet induced-obese mice, with hyperinsulinemia) which contribute to preserving vascular function [112]. Evidence of augmented blood flow at early stages of diabetes may also reflect higher NO• production [102, 113].

An upregulated NOSs expression/activity may explain the elevated NO• production in the initial stages of T2D. In the aorta of GK rats, increased eNOS mRNA expression was observed in young (12-week-old) compared to age-matched controls, whereas eNOS, iNOS, and nNOS mRNAs were lower in older rats (70-week old) compared to the younger (12-week old) rats [114]. Acute exposure of ET to high-glucose concentrations upregulates eNOS expression via activation of PKC [115]. Both eNOS and iNOS gene expressions were up-regulated in ECs in short-term exposure to high-glucose concentrations (10 and 50 mM), resulting in increased NO• concentration in the media (from 8 to 11.5 and 12.5 µM) [98]. An up-regulated eNOS expression in the ET of pre-glomerular and post-glomerular vessels was also evident in the early stages of diabetes in rats [116].

Different mechanisms are involved in decreased vascular NO• production in established diabetes (Fig. 1), including (1) decreased eNOS activity [117] and expression [118], (2), increased arginase activity [83, 119], and l-arginine deficiency [83], and (3) decreased eNOS sensitivity to ACh [120]. Due to decreased activity and expression of eNOS, diabetic patients displayed a decreased conversion of l-arginine to NO•, and ET of human diabetic vessels cannot generate enough NO• to regulate blood flow [10, 100]. Decreased vascular eNOS activity/expression in T2D is due to (A) increased dimethylarginine dimethylaminohydrolase (DDAH) activity, which increases asymmetric dimethyl arginine (ADMA) [117], (B) increased AEGs, which decreases eNOS mRNA stability and eNOS phosphorylation [118], (C) increased NOX, which uncouples eNOS and oxidizes BH_4_ [121], (D) impaired insulin signaling [122], (F) increased arginase activity [120], and (G) decreased NOS sensitivity to ACh [120].

During the establishment of T2D, DDAH (the enzyme that metabolizes the endogenous competitive inhibitor of NOS enzymes, ADMA) activity decreased by about 44% in the abdominal aorta of T2D rats. Incubation of human ET cells and rat VSMCs in high-glucose resulted in ADMA accumulation, decreased eNOS activity, and reduced cGMP levels [117]. Hyperglycemia inhibits ET-eNOS activity through post-translational modification, that is, by increasing O-linked N-acetylglucosamine modification of eNOS and decreasing phosphorylation at O-linked serine residue 1177 [123]. Prolonged exposure of vascular ET with AGEs (i.e., glucose-derived moieties that are produced non-enzymatically through glycation reaction between glucose and the amino groups of proteins) under hyperglycemic conditions significantly reduce eNOS expression, eNOS mRNA stability, and eNOS phosphorylation (at Ser^1177^) and its activity, and cellular NO• levels [118].

T2D also initiates a cascade of events in the vessel wall, including NOX-induced ROS over-production, oxidation of BH_4_, and uncoupling of eNOS in vascular ET, leading to decreased NO• availability [121]. In addition, T2D impairs phosphatidylinositol 3-kinase (PI3K)-protein kinase B (PKB/Akt) insulin signaling pathway in the vascular ET; this pathway stimulates eNOS and thus NO production; therefore, its impairment decreases NO• production in T2D vessels [122].

High-glucose concentrations in vascular ET caused a 66.7% increase in arginase activity, leading to a 27% decreased NO• production [119]. Furthermore, elevated circulating free heme in T2D impairs l-arginine transport across RBC membranes, increases l-arginine consumption by arginase, and reduces l-arginine availability for NO• production by RBC [124]; in this state, RBCs tend to catabolize l-arginine to ornithine, citrulline, and urea [125]. Elevated RBC-arginase activity in patients with T2D causes decreased NO• bioavailability and ET dysfunction [126].

A progressive decreased eNOS sensitivity to ACh was also shown to be worsened with the diabetes duration; EC5_0_ for ACh in diabetic arteries was increased from 13.5 nM after 6 weeks to 63 and 100 nM after 16 and 24 weeks of diabetes [120].

## NO• storage pool within VSM

VSMC is proposed to contain a NO• storage pool comprised of NO• and NO•-equivalents, including NO_2_^–^, SNOs [i.e., *S*-nitrosocysteine (cysNO), GSNO], and dinitrosyl iron complexes (DNICs, a non-heme-iron nitrosyl species). NO• and NO•-equivalents enter the VSMCs from different sources, including ET (in response to shear stress and agonists like ACh), pVAT, nerve fibers, mast cells, and circulation [127, 128]. The predicted NO• concentration in the VSM ranges from 20–100 pM to 400 nM [129]. However, depending on the vessel diameter, NO•-RBC reaction rate constant (*K*_NO•-RBC_), and blood flow velocity, NO• concentration in the VSM may reach up to ~1100 nM (e.g., 281–1163 nM for 50 μm arteriole over the blood velocity range of 0.5-4.0 cm/s and *K*_NO•-RBC_ of 0.2 × 10^5 ^M/s) [93].

**Fig. 2:**
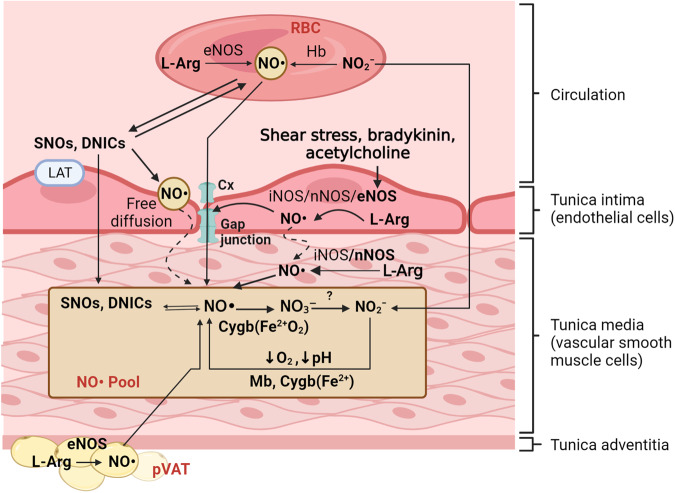
Main sources of nitric oxide (NO•) pool within the vascular smooth muscle cells (VSMCs). The pool of NO• within the VSMCs is comprised of NO synthase (NOS)-derived NO• [produced within VSMC, transporting from endothelial cells, perivascular adipose tissue (pVAT) or other vascular components], and NO•/NO•-equivalents [i.e., nitrite (NO_2_^–^), *S*-nitrosothiols [SNOs, i.e., *S*-nitrosocysteine (cysNO), nitrosoglutathione (GSNO), dinitrosyl iron complexes (DNICs, a non-heme-iron nitrosyl species)] that are derived from red blood cells (RBCs) and other circulating NO• sources. Cygb, cytoglobin; eNOS, endothelial NOS; Hb, hemoglobin; iNOS, inducible NOS; l-Arg, l-arginine; LAT, L-type amino acid transporter; Mb, myoglobin; nNOS, neural NOS.

A diffusion constant of 3.3 × 10^−5^ cm^2^/s in all directions to a distance of 150–600 μm enables NO• to diffuse from its sources simply (e.g., ET, pVAT, or circulation) to VSMCs [80, 128] (Fig. 2). Connexins (Cx37, Cx40, Cx43, Cx46) are involved in NO• diffusion across the plasma membranes of ET [130]; *S*-nitrosylation of cysteine residues of connexins by NO• derivates activates the opening of the connexins [131]. The expression pattern and activity of connexins are changed in T2D [132, 133]; thus, decreased vascular NO• availability can be attributed partly to impaired delivery of circulating NO• to the vessel wall.

Circulating NO• penetrates the vessel wall despite very effective scavenging by Hb. The RBC-free layer near the vascular ET reduces the rate of free NO• trapping by RBCs-Hb. It allows it to escape scavenging by Hb and reach the VSM in physiologically significant concentrations to induce vasorelaxation [134]. The NO• delivery from circulating sources into the vessel wall is governed by a dynamic cycle between circulatory Hb-NO and Hb-SNO [95], enabling RBCs to act as SNOs reactor, regulating plasma SNOs levels [135] and delivery of NO• to its target [95, 136]. This dynamic cycle is impaired in hyperglycemic conditions like T2D; glycosylated RBCs are likely to be dysfunctional compared to normal-glycosylated RBCs [137]. NO• is trapped within the glycosylated-Hb and cannot be transferred into the vascular cells, a condition that results in a reduced NO• bioavailability within the diabetic vessels; compared to normal-glycosylated Hb, NO• mainly exists in the form of Hb-NO• (0.044 *vs*. 0.013 percent NO• per Hb mol/L) rather Hb-SNO (0.025 *vs*. 0.032 percent NO/Hb mol/L) within the highly-glycosylated RBCs (indicated as HbA1C > 10.7%) [137]. The rate of NO• release from Hb-NO• is extremely low [138].

Cellular DNICs [i.e., Fe(RS)_2_(NO•)_2_ complexes produced via interaction of NO• with iron-sulfur clusters of proteins] deliver NO• into the cytosol of VSMC; they are important NO• storage form in the VSMC [139]. DNICs and SNOs are suggested to release NO• outside the VSMC (because they are membrane impermeable) [127]; however, selective transport of SNOs via *L*-type amino acid transporters (i.e., LAT1 and LAT2, in vascular ET and SMCs) [140], and dipeptide transporters (PEPT2) (as documented in other cells like macrophages) [141] may occur. Handoff of NO• from extracellular SNOs to the plasma membrane and VSM thiols via transnitrosylation with subsequent transport of the NO• to the cytoplasm has also been proposed [127].

The NO• storage pool in VSMC seems to release NO• in a controlled manner upon extracellular stimulations [127]. NO• release from the VSMC store (DNICs and SNOs) is light- [142] and thiol-[143] sensitive. NO_2_ likely contributes to the NO• stores via conversion into NO•, probably via the action of metalloproteins, including Hb, myoglobin (Mb), cytoglobin ([Cygb), xanthine oxidase, cytochrome c oxidase, and eNOS, that is favored by hypoxia and low pH [127]. Cygb [i.e., a globin expressed at μM levels (~3.5–5.0 μM [144]) and co-localized with myosin heavy chain [145]] regulates NO• bioavailability within the VSM [146]; under a normal O_2_ level, Cygb metabolizes excessive amount of NO• by dioxygenation (converting NO• to NO_3_, a rate of 11.6 ± 0.6 nM/s in mouse aorta); in contrast, under a hypoxic condition, it generates NO• from NO_2_ (referred to as O_2_-dependent NO•-dioxygenase and NO_2_-reductase, respectively) [146], and that NO• binds to sGC in the VSM, making the vessel to be relaxed [147]. About 78% of NO• metabolism in VSMCs is Cygb-dependent [144]. In human VSMCs, Cygb-mediated NO• production from NO_2_ (at the physiological intracellular level of NO_2_ ~ 10 μM) is estimated to be ~7 and 35 pM/s in VSM, at pH 7.0 and 5.5, respectively); an amount that can rise to 10-fold ( ~ 350 pM/s) under acidic condition (pH=5.5) and chronic hypoxia (i.e., intracellular Cygb concentration of ~350 μM) [147]. In human VSMCs, Cygb-mediated NO• release corresponds to about 40% of cGMP activation under hypoxic conditions [147].

VSMCs Mb is another important vascular NO_2_ reductase; deletion of Mb significantly decreased NO_2_-NO•-cGMP mediated vasorelaxation (~57% decrease in cGMP production, from 1300 to 550 fmol/mg in mice aorta), indicating that Mb is also a bioconvertor of NO_2_ to NO• in VSM [148]. Chronic hyperglycemia, resulting in a non-enzymatic reaction of glucose with an amino group of Mb and changes in the Mb’s structure and function, may decrease NO• availability in the VSM in T2D [149].

## NO• actions in VSM in normal conditions and T2D

### Role of NO• in VSM contraction

**Fig. 3:**
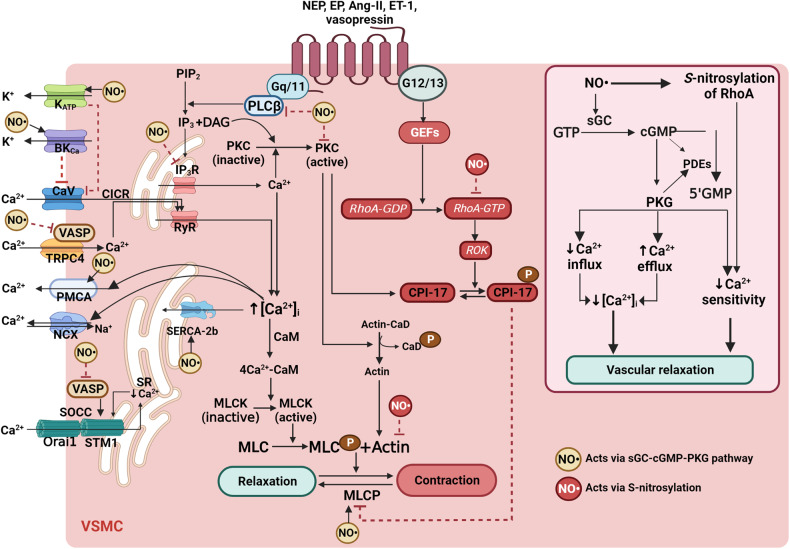
The main mechanisms of vascular smooth muscle (VSM) contraction and underlying mechanisms of NO•-mediated VSM relaxation. VSM contraction is initiated by increasing [Ca^2+^]_i_ via an influx of Ca^2+^ from extracellular fluid Ca^2+^ channels (i.e., voltage-dependent, receptor-operated, transient receptor potential (TRP), store-operated, and stretch-activated channels) or Ca^2+^ release from the sarcoplasmic reticulum (via PLC-IP_3_-IP_3_R pathway and Ca^2+^-induced Ca^2+^release pathway via RyR). Cytosolic Ca^2+^ binds to CaM, and the Ca^2+^–CaM complex activates MLCK, which phosphorylates MLC and causes contraction. Increased force of VSM contraction is mediated via Rho-Rho kinase (ROK)- and PKC-dependent MLCP inhibition. As indicated in the right section, NO• signaling in the VSM includes two major pathways, i.e., NO•-sGC-cGMP-PKG pathway and protein *S*-nitrosylation of target proteins. The downstream cGMP-dependent pathways may be Ca^2+^-dependent (i.e., decreasing intracellular Ca^2+^ and Ca^2+^ desensitization) or Ca^2+^-independent. The cGMP-dependent pathway decreases Ca^2+^ influx and increases Ca^2+^ efflux from the cytosol of VSM. NO• decreases Ca^2+^ sensitivity by phosphorylating MLCP, thereby increasing MLCP activity, reducing agonist-induced Ca^2+^ sensitization of contraction, and inhibiting PKC activation; PKC promotes contraction of VSM by phosphorylating CaD, which in its non-phosphorylated form inhibits actin-myosin interaction, and by inhibiting MLCP and thereby increase in Ca^2+^ sensitivity. S-nitrosylation of RhoA, inhibiting the Rho-ROK-MLCP pathway, is the cGMP-independent pathway for NO•-induced vasodilation. BK_Ca_; Ca^2+^-activated K^+^-channels; CaM, calmodulin; CaD, caldesmon; CaV, voltage-gated Ca^2+^channels; DAG; diacylglycerol; GEFs, guanine nucleotide exchange factors; GTP, guanosine triphosphate; GDP, guanosine diphosphate; IP3, inositol triphosphate; IP3R, IP3 receptor; MLCP, myosin light chain phosphatase; MLC, myosin light chain; MLCK myosin light chain kinase; NCX, Na^+^–Ca^2+^ exchanger; PDE, phosphodiesterase enzyme; PKC, protein kinase C; PKG, protein kinase G; sGC, soluble guanylate cyclase; PLC, phospholipase C; PMCA, plasma membrane Ca^2+^-ATPase; RYR, ryanodine receptors; SERCA, sarcoplasmic/endoplasmic reticulum Ca^2+^-ATPase; TRPC4, transient receptor potential channel 4; VASP; vasodilator-stimulated phosphoprotein.

Mechanisms underlying VSM contraction are illustrated in Fig. 3. Signal transduction of NO• in VSM involves two major pathways: (1) the indirect pathway of NO•-sGC-cGMP-PKG [128, 150, 151] and (2) the direct pathway of protein *S*-nitrosylation [128, 150, 151], also called cGMP-dependent and -independent pathways, respectively [152] **(**Fig. 3**)**. Although both pathways are found in the VSM, NO•-mediated vasorelaxation is mostly cGMP-dependent at a normal oxygen pressure [152]. The NO• concentrations eliciting a physiological response in VSM have been estimated to fall from 100 pM to 5 nM [153].

At low nM concentrations, NO• binds to ferrous heme residue (the NO•-biding site) of its own receptor, sGC, and activates it [150, 154]. Activated sGC converts GTP to second messenger cGMP, which leads to tissue-specific biological effects of NO• [128, 150]. cGMP exerts its effects in VSMCs by cGMP-dependent protein kinase (PKG, or cGK) and by affecting cGMP-binding phosphodiesterase [154]. PKG, the principal mediator of cGMP-induced VSM relaxation, acts via Ca^2+^-dependent mechanisms, i.e., decreasing [Ca^2+^]_i_ [152, 154, 155] and Ca^2+^ desensitization [152, 154], as well as Ca^2+^-independent mechanisms [156].

NO•-sGC-cGMP-PKG pathway decreases Ca^2+^ influx and increases Ca^2+^ efflux from the cytosol of VSMCs. PKG increases the activity of big conductance Ca^2+^-activated K^+^-channels (BK_Ca_), and cGMP activates ATP-sensitive K^+^ (K_ATP_) channels in VSMCs [157], both of which hyperpolarize cell membrane and inhibit Ca^2+^ entry through voltage-gated Ca^2+^channels (VGCCs or CaV) [154, 155]. cGMP inhibits PLC and IP_3_ generation [154]; in addition, PKG phosphorylates the IP_3_ receptor and decreases its activity, leading to decreased Ca^2+^ release from the SR [154]. Furthermore, PKG increases Ca^2+^ efflux by activating PMCA and SERCA [154].

In the case of Ca^2+^ desensitization, PKG phosphorylates MBS of MLCP, thereby increasing MLCP activity and reducing agonist-induced Ca^2+^ sensitization [154, 155]. In addition, cGMP-PKG inhibits PKC activation [154]. PKC promotes contraction of VSM by phosphorylating caldesmon (CaD), which in its non-phosphorylated form inhibits actin-myosin interaction, and by inhibiting MLCP and thereby increasing Ca^2+^ sensitivity [154].

Vasodilator-stimulated phosphoprotein (VASP), a downstream molecule of NO• signaling, is found in VSMCs [158] and is phosphorylated at serine 239 by PKG [158, 159]. In rabbit thoracic aorta, SNP increases Ser239-phosphorylated VASP, and *L*-*N*^G^-Nitro arginine methyl ester (*L*-NAME) and 1h-Oxadiazoloquinoxalin-1-one (ODQ) decrease it by about 80% and 85%, respectively, indicating dependency of this phosphorylation to NO•-cGMP-PKG pathway [158]. However, the role of VASP in regulating vascular tone remains to be elucidated [158]. Aortic rings of wild-type and VASP null mice showed similar relaxant responses to cGMP, ACh, and SNP, indicating that VASP is not essential for vasodilator-induced relaxation of VSM [160]. However, in mesangial cells, SNP via the NO•-cGMP-PKG-Iα pathway increases Ser239-phosphorylated VASP and inhibits store-operated Ca^2+^ entry [161]. In addition, in mesenteric arteries of Sprague-Dawley rats, incubation of mesenteric vessels with NONOate, a NO• donor, caused Ser239 phosphorylation and colocalization of VASP with TRP channel 4 (TRPC4); 1,1-Diethyl-3-oxotriazane-2-ol (NONOate) and 8p-CPT-cGMP also blocked cyclopiazonic acid (CPA, a selective inhibitor of SERCA)-induced increase in Ca^2+^ entry [159]. It has been suggested that Ser239-phosphorylated VASP decreases Ca^2+^ entry through SOCCs in VSMCs and leads to vasorelaxation [159].

Protein S-nitrosylation, the binding of a NO• moiety to a thiol group of a cysteine residue to form SNO [128], is a specific, reversible, and enzymatic reaction leading to specific protein modification [151, 162–164]. For example, in rat aortic VSMCs, S-nitrosylation of RhoA by S-nitrosocystein inhibits the Rho-ROK-MLCP pathway, suggesting a cGMP-independent pathway for vasodilation [165]. In addition, S-nitrosylated actin at Cysteine-374 produces a dose-dependent high potency (EC_50_ ≈ 9.37 nM) relaxation in rat abdominal aorta strips, which may be sGC-dependent or independent [166].

### The underlying mechanisms of impaired NO• action on VSM in T2D

**Fig. 4:**
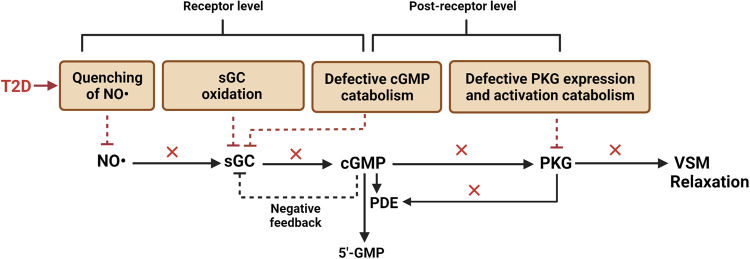
Proposed underlying mechanisms of vascular smooth muscle (VSM) hypo-responsiveness to nitric oxide (NO•) in type 2 diabetes (T2D). The vascular hypo-responsiveness to NO• is evident at the receptor level (soluble guanylate cyclase, sGC) and post-receptor level (sGC-downstream pathway). Mechanisms acting at the receptor level include: (a) quenching of NO• by advanced glycation end products (AGEs) and reactive oxygen species (ROSs), and (b) sGC desensitization via ROS-induced sGC oxidation, and augmented cGMP-induced negative feedback on sGC [because of reduced phosphodiesterase (PDE) activity and increased cGMP level]. Mechanisms acting at the post-receptor level include (a) defective cGMP catabolism and (b) hyperglycemia-induced downregulation of protein kinase G (PKG).

The main cause of vascular hypo-responsiveness to NO• is impairment of the main physiologically relevant NO• signaling cascade, i.e., the NO•-cGMP-PKG pathway; this may occur at the receptor level (i.e., quenching of NO• and/or sGC desensitization [53, 167]) and post-receptor level [i.e., cGMP generation by sGC, cGMP degradation by phosphodiesterase (PDE), and/or cGMP-induced activation of PKG] [14, 18]. The T2D-induced mechanisms of impaired NO• action on VSM relaxation are illustrated in Fig. 4.

The NO• bioavailability (extent NO• becomes available to its targets) reduces in diabetic vessels regardless of high NO• production at early stages of diabetes [115, 121], evidence indicating NO• quenching. In hyperglycemic states, high levels of AGEs are accumulated within the subendothelial collagen, quench NO• activity and impair its vasodilatory effect [168]. Quenching occurs by a direct reaction between the NO• and the AGEs within less than 5 seconds [168]. The apparent paradox of diabetes-induced eNOS upregulation at early stages concomitantly with a decreased vascular NO• bioavailability is attributed to the elevated formation of superoxide anion O_2_^• –^ and peroxynitrite (ONOO^-^) by vascular ET [121]. Furthermore, endothelial NOX (NOX1, NOX2, and NOX5), potent stimulators of ROS production in ECs in hyperglycemia, reduce NO• availability by its conversion to ONOO^-^ [169, 170]. Incubation of human umbilical vein endothelial cells (HUVEC) in high-glucose media resulted in upregulated NO• production (~2-3 fold), decreased NO• availability by 52%, and increased ONOO^-^ levels by 240% within 30 min [170]. Other ROS generators (e.g., mitochondria, uncoupled eNOS, xanthine oxidase, and cyclooxygenase-1) under hyperglycemic conditions may accelerate NO• quenching within the diabetic vessels [171].

Desensitization of sGC is the transition to a state in which sGC response to a new NO• molecule is reduced or abolished [172]. Both the potency and efficacy of NO• to activate sGC (i.e., measured as cGMP production) were lower in the aorta of GK rats [167] and T2D patients [173]. In addition, SNP-induced increase in cGMP was lower in VSMCs from insulin-resistant obese Zucker rats (OZR) than controls; this response was maintained in the presence of IBMX (unselective PDE inhibitor), indicating the impaired ability of NO• to activate sGC [18]. In addition, it was not due to different protein expressions of sGC subunits (α1 and β1) [18]. These data indicate sGC desensitization as an underlying mechanism for vascular NO• resistance in T2D.

Although not entirely determined, two mechanisms seem to result in sGC desensitization: 1) ROS-induced sGC oxidation, and 2) defective cGMP catabolism. The binding of NO• to reduced ferrous (Fe^2+^) heme residue of sGC increases its catalytic activity and cGMP production from GTP. The reduced sGC response to NO• seems to be due to the reduced enzyme’s heme content and/or oxidation of the heme iron under hyperglycemic conditions because the expression of sGC was reported to be preserved [167] or even increased in the diabetic vessels [53]. High levels of ROS can oxidize the sGC heme iron to the ferric form (Fe^3+^), rendering sGC insensitive to normal levels of NO• and developing NO• resistance [174, 175]. Indeed, one of the heterodimeric redox-sensitive sites of sGC is a prosthetic heme group bound to the β subunit, and oxidation of its ferrous heme inhibits the NO•-mediated activation of the receptor [176]. Heme-dependent hypo-responsiveness of sGC to NO• is supported by evidence showing that response to BAY 41-2272 (an sGC stimulator, i.e., can bind directly to the reduced-form of heme-containing sGC) was reduced in diabetic resistance arteries, while heme-independent sGC activation (using BAY 58-2667, an sGC activator, i.e., binds directly to oxidized-form of heme-containing sGC) was relatively preserved [53]. Likewise, other vascular beds, e.g., aorta derived from diabetic GK rats and isolated vessels from T2D humans, exhibited a preserved and enhanced relaxation response to sGC activators (i.e., heme-independent activator protoporphyrin-IX and BAY 58–2667, respectively) [167, 177]. This phenomenon has been introduced as a sub-phenotype of ET dysfunction, characterized by NO• resistance at the receptor level in the blood vessels of patients with T2D [13, 177].

Defective cGMP catabolism may also contribute to sGC desensitization in diabetic vessels. PDEs control the abundance of cGMP [128]. Among 11 families of PDE that have been identified [128], PDE1, PDE3, and PDE5 degrade cGMP in VSM [156], and PDE5 is the main one involved in cGMP catabolism in the smooth muscle [178]. cGMP binds to PDE5 and increases its activity which means PDE5 activity increases following increased cGMP production [156]. In addition, PKG-I phosphorylates PDE5 and prolongs its activation by increasing its affinity for cGMP [156]. This provides a negative feedback mechanism to preserve the sensitivity of the NO•-sGC-cGMP-PKG signaling pathway [156]. In VSMCs isolated from the aorta, baseline cGMP concentrations were about 3 times (1.83 ± 0.08 *vs*. 0.67 ± 0.07 pmol/mg protein) higher in insulin-resistant-obese Zucker rats than controls [18]. This was not affected by the NOS inhibitor *L*-NMMA, which rules out sGC hyperactivation [18]. In addition, sGC inhibitors (ODQ and Ambroxol) decreased baseline cGMP in VSMCs from OZR but not controls. Still, baseline cGMP remained higher in VSMCs from OZR, suggesting defective cGMP catabolism [18]. Baseline PDE5 activity was lower in VSMCs from OZR, and the IBMX-induced increase in cGMP was smaller in VSMCs from OZR than in controls, suggesting defective PDE activity in VSMCs from OZR [18].

Some evidence supports the notion that vascular hypo-responsiveness to NO• is at least in part due to post-receptor events in the NO•-sGC-cGMP-PKG signaling pathway. Hyperglycemia downregulates mRNA and protein levels of PKG-I in VSMCs through altered NOX signaling [179]. In diabetic vessels, the ability of cGMP to activate PKG and PKG-dependent activation of PDE5 are also impaired [18]. No further evidence is available regarding T2D-induced changes in sGC downstream signaling.

## Conclusion and perspectives

Both animal and human studies provide evidence for the presence of vascular NO• resistance in T2D patients, which is an independent risk factor for cardiovascular events. Human studies indicate a 13-94% decrease in ET-dependent VSM relaxation and a 6–42% decresae in the vasodilatory action of exogenous NO• in different vascular beds of patients with T2D (see Table 1). Vascular NO• resistance in T2D is stage-dependent and displays a progressive spectrum, initially manifested by an augmented or preserved vascular NO• production and/or VSM response to NO•, followed by a reduced NO• bioavailability and/or partial to almost entirely impaired NO• function in VSM, in both the macro- and microvessels. Because various vessels may have different capacities of NO• production [180] and heterogeneously respond to vasodilatory action of NO• (probably due to their different morphology, functions, and diverse receptor and ion channel populations) [181–183], the vascular NO• resistance seems to be progressed in a time-course manner with a different magnitude in the vessels. Although it needs to be proved, small coronary arteries seem to be affected by T2D-induced vascular NO• resistance earlier and to a greater extent compared to mesenteric resistance arteries and large elastic vessels like the aorta [64]. These observations may imply that different vessels probably exhibit diverse phenotypes of NO• resistance in T2D.

The mechanisms underlying vascular NO• resistance in T2D are not fully understood; however, a leading hypothesis proved in several vascular beds is that hyperglycemia-induced overproduction of ROS is a key player initiating a cascade of events (i.e., polyol and hexosamine pathway, AGEs production, induction of PKC-dependent pathways and NOX activity) in the vessel wall resulting in decreased NO• availability (by inhibiting NOS expression/activity and quenching NO• activity), sGC desensitization (by oxidizing its heme residue, and/or nitrosylating its cysteine residues) and impaired cGMP-PKG pathway (by decreasing cGMP catabolism, and/or inhibiting PKG expression/activity) in VSM.

New pharmacological approaches that upregulate vascular NO• availability, re-sensitize or bypass the non-responsive pathways to NO•, and target key vascular sources of ROS production are potentially relevant strategies in preventing and retarding the progression of T2D-induced vascular NO• resistance. Beneficial effects of some pharmaceutical agents, including angiotensin-converting enzyme inhibitors (e.g., ramipril, perindopril), the anti-anginal agent perhexiline, insulin (by decreasing oxidative stress and superoxide production), statins (by upregulating eNOS expression and activity), NO_2_, and sGC activators, have been documented for attenuating vascular NO• resistance [14, 15]. Furthermore, the use of nitroxyl donors, e.g., Angeli’s salt, is now proposed as an effective strategy for overcoming NO• resistance in T2D [15] because of its antioxidant properties and potential ability to target alternative biological molecules [e.g., calcitonin gene-related peptide (CGRP), thiol residues] and distinct pathways [i.e., cyclic AMP(cAMP)-protein kinase A (PKA), rather than cGMP-PKG] involved in vasodilation.

Quantifying the contribution of the impaired pathways (i.e., reduced NO• synthesis/availability, sGC desensitization, and impaired cGMP-PKG) and determining their chronology in developing vascular NO• resistance in T2D may drive therapeutic approaches to design specified and timely interventions. Furthermore, considering the diverse phenotypes of NO• resistance in different vessels may help develop specific vessel-targeted drug delivery platforms to overcome vascular NO• resistance in T2D. Concurrent management of hyperglycemia, insulin resistance, and oxidative stress is also essential to ameliorate vascular NO• resistance in T2D patients.

## Supplementary information

Supplementary Table 1

Legend of supplementary table
