# Supplementary material for: Vascular nitric oxide resistance in type 2 diabetes
Source: Cell Death Dis. 2023 Jul 11;14(7):410. doi: 10.1038/s41419-023-05935-5 (PMC10336063; doi:10.1038/s41419-023-05935-5)
Supplement: Supplementary file 1 — Supplementary Table 1 [file 41419_2023_5935_MOESM1_ESM.docx]

| **Supplementary Table 1.** Expression/activity of nitric oxide (NO•) synthase (NOS) isoforms in different components of the vessels’ wall in animals and humans. NOSs expression and NO• in the endothelium (ET) is site-specific (in the different hierarchy of the vessels) and has a considerable interspecies variation ([1](#_ENREF_1)); endothelial NOS (eNOS) seems to be the predominant isoform in the perivascular adipose tissue (pVAT) ([2](#_ENREF_2), [3](#_ENREF_3)), while vascular smooth muscle (VSM) mainly expresses neural NOS (nNOS) ([2](#_ENREF_2), [4](#_ENREF_4)). | | | | | |
| --- | --- | --- | --- | --- | --- |
| **Species** | **Vessel type** | **Vessel component** | **Expression/activity of NOS isoforms** | | |
|  |  |  | eNOS | nNOS | iNOS |
| Pigs ^(^[^5^](#_ENREF_5)^)^ | Carotid artery | ET, VSM | + | + | + |
| Rats ^(^[^6^](#_ENREF_6)^)^ | Carotid artery | ET | + | + | - |
| Rats ^(^[^6^](#_ENREF_6)^)^ | Carotid artery | VSM | + | ++ | - |
| Rats ^(^[^6^](#_ENREF_6)^)^ | Carotid artery | Adventitia | NR | + | NR |
| Rats ^(^[^7^](#_ENREF_7)^)^ | Brain and mesenteric arterioles and venules | ET | + | + | NR |
| Rats ^(^[^7^](#_ENREF_7)^)^ | Mesenteric arterioles and venules | Mast cells | Little or no | + | Little or no |
| Rats ^(^[^8^](#_ENREF_8)^)^ | Pulmonary artery | VSM | NR | + | NR |
| Rats ^(^[^9^](#_ENREF_9)^)^ | Abdominal aorta, thoracic aorta | pVAT | + | NR | NR |
| Rats ^(^[^10^](#_ENREF_10)^)^ | Thoracic aorta | pVAT | + | NR | NR |
| Newborn rats ^(^[^8^](#_ENREF_8)^)^ | Coronary and pulmonary arteries | ET | NR | + | NR |
| Hamsters ^(^[^3^](#_ENREF_3)^)^ | Cheek pouch arterioles and venules | ET | + | - | - |
| Mice ^(^[^11^](#_ENREF_11)^)^ | Aorta | pVAT | + | + | + |
| Mice ^(^[^12^](#_ENREF_12)^)^ | Thoracic aorta | pVAT | + | NR | NR |
| Humans ^(^[^5^](#_ENREF_5)^)^ | Arteria | ET | + | - | - |
|  | Pancreas artery | VSM | - | + | - |
|  | Arterioles | ET, VSM | + | + | + |
|  | Venous blood vessel of the pancreas | ET, VSM | + | + | + |
|  | Muscular type arteries | VSM | + | + | + |
|  | Aorta | VSM | - | + | - |
|  | Arteria mammaria | VSM | + | + | + |
|  | Arteria mammaria | ET | + | - | - |
| Humans ^(^[^13^](#_ENREF_13)^)^ | Aorta | VSM | - | + | - |
| Humans ^(^[^14^](#_ENREF_14)^)^ | Aorta, larger arteries | ET, vasa vasorum in adventitia and media | + | - | - |
|  | Aorta, larger arteries | VSM | - | NR | NR |
|  | Atherosclerotic arteries | ET | +  (↓compared to the normal aorta) | + | + |
|  | Atherosclerotic carotid arteries | Macrophages, mesenchymal-appearing intimal cells |  | + | + |
| Humans ^(^[^15^](#_ENREF_15)^)^ | Capillaries, arteries, and arterioles in the lung, liver, skin, and umbilicus | ET | NR | + | NR |
| Humans ^(^[^16^](#_ENREF_16)^)^ | Lung capillaries | ET | NR | + | NR |
| Humans ^(^[^17^](#_ENREF_17)^)^ | Saphenous vein | pVAT | ++ | + | + |
| ++ most abundant isoform; iNOS, inducible NOS; NR, not reported. | | | | | |

1. Geiger M, Stone A, Mason SN, Oldham KT, Guice KS. Differential nitric oxide production by microvascular and macrovascular endothelial cells. The American journal of physiology. 1997;273(1 Pt 1):L275-81.

2. Schwarz PM, Kleinert H, Förstermann U. Potential functional significance of brain-type and muscle-type nitric oxide synthase I expressed in adventitia and media of rat aorta. Arterioscler Thromb Vasc Biol. 1999;19(11):2584-90.

3. Figueroa XF, Martínez AD, González DR, Jara PI, Ayala S, Boric MP. In vivo assessment of microvascular nitric oxide production and its relation with blood flow. American Journal of Physiology-Heart and Circulatory Physiology. 2001;280(3):H1222-H31.

4. Cheah LS, Gwee M, Das R, Ballard H, Yang YF, Daniel EE, et al. Evidence for the existence of a constitutive nitric oxide synthase in vascular smooth muscle. Clinical and experimental pharmacology & physiology. 2002;29(8):725-7.

5. Buchwalow IB, Podzuweit T, Bocker W, Samoilova VE, Thomas S, Wellner M, et al. Vascular smooth muscle and nitric oxide synthase. FASEB journal : official publication of the Federation of American Societies for Experimental Biology. 2002;16(6):500-8.

6. Boulanger CM, Heymes C, Benessiano J, Geske RS, Lévy BI, Vanhoutte PM. Neuronal nitric oxide synthase is expressed in rat vascular smooth muscle cells: activation by angiotensin II in hypertension. Circulation research. 1998;83(12):1271-8.

7. Kashiwagi S, Kajimura M, Yoshimura Y, Suematsu M. Nonendothelial Source of Nitric Oxide in Arterioles But Not in Venules. Circulation Research. 2002;91(12):e55-e64.

8. Loesch A, Burnstock G. Ultrastructural localization of nitric oxide synthase and endothelin in coronary and pulmonary arteries of newborn rats. Cell and tissue research. 1995;279(3):475-83.

9. Victorio JA, Fontes MT, Rossoni LV, Davel AP. Different Anti-Contractile Function and Nitric Oxide Production of Thoracic and Abdominal Perivascular Adipose Tissues. Frontiers in Physiology. 2016;7.

10. Araujo HN, Valgas da Silva CP, Sponton AC, Clerici SP, Davel AP, Antunes E, et al. Perivascular adipose tissue and vascular responses in healthy trained rats. Life sciences. 2015;125:79-87.

11. Xia N, Horke S, Habermeier A, Closs EI, Reifenberg G, Gericke A, et al. Uncoupling of Endothelial Nitric Oxide Synthase in Perivascular Adipose Tissue of Diet-Induced Obese Mice. Arteriosclerosis, Thrombosis, and Vascular Biology. 2016;36(1):78-85.

12. Baltieri N, Guizoni DM, Victorio JA, Davel AP. Protective Role of Perivascular Adipose Tissue in Endothelial Dysfunction and Insulin-Induced Vasodilatation of Hypercholesterolemic LDL Receptor-Deficient Mice. Frontiers in Physiology. 2018;9.

13. Papadaki M, Tilton RG, Eskin SG, McIntire LV. Nitric oxide production by cultured human aortic smooth muscle cells: stimulation by fluid flow. The American journal of physiology. 1998;274(2 Pt 2):H616-26.

14. Wilcox JN, Subramanian RR, Sundell CL, Tracey WR, Pollock JS, Harrison DG, et al. Expression of multiple isoforms of nitric oxide synthase in normal and atherosclerotic vessels. Arterioscler Thromb Vasc Biol. 1997;17(11):2479-88.

15. Springall DR, Riveros-Moreno V, Buttery L, Suburo A, Bishop AE, Merrett M, et al. Immunological detection of nitric oxide synthase(s) in human tissues using heterologous antibodies suggesting different isoforms. Histochemistry. 1992;98(4):259-66.

16. Lührs H, Papadopoulos T, Schmidt HH, Menzel T. Type I nitric oxide synthase in the human lung is predominantly expressed in capillary endothelial cells. Respiration physiology. 2002;129(3):367-74.

17. Dashwood MR, Dooley A, Shi-Wen X, Abraham DJ, Souza DSR. Does Periadventitial Fat-Derived Nitric Oxide Play a Role in Improved Saphenous Vein Graft Patency in Patients Undergoing Coronary Artery Bypass Surgery? Journal of Vascular Research. 2007;44(3):175-81.
