# Supplementary material for: Vascular nitric oxide resistance in type 2 diabetes
Source: Cell Death Dis. 2023 Jul 11;14(7):410. doi: 10.1038/s41419-023-05935-5 (PMC10336063; doi:10.1038/s41419-023-05935-5)
Supplement: Supplementary file 2 — Legend of supplementary table [file 41419_2023_5935_MOESM2_ESM.docx]

**Legend of supplementary table**

**Supplementary Table 1.** Expression/activity of nitric oxide (NO•) synthase (NOS) isoforms in different components of the vessels’ wall in animals and humans. NOSs expression and NO• in the endothelium (ET) is site-specific (in the different hierarchy of the vessels) and has a considerable interspecies variation ([1](#_ENREF_1)); endothelial NOS (eNOS) seems to be the predominant isoform in the perivascular adipose tissue (pVAT) ([2](#_ENREF_2), [3](#_ENREF_3)), while vascular smooth muscle (VSM) mainly expresses neural NOS (nNOS) ([2](#_ENREF_2), [4](#_ENREF_4)).
